# Utilizing Ripple Effects Mapping to Assess the Impact of an Undergraduate Global Health Program

**DOI:** 10.5334/aogh.3933

**Published:** 2023-01-20

**Authors:** Talia Bailes, Mia Haller, Jeanne Moseley

**Affiliations:** 1 Cornell University, US; 2 Department of Public & Ecosystem Health, MPH Program, Cornell University, US

**Keywords:** Global Health Education, Public Health Education, Ripple Effects Mapping, Undergraduate

## Abstract

**Background:**

In the last ten years, there has been a dramatic rise in student interest in global health as an academic discipline and an increase in academic offerings in the field at both the undergraduate and graduate levels. However, information is limited on the impact of global health programming on students, alumni, and partners involved.

**Objective:**

The objective of this study was to utilize Ripple Effects Mapping (REM) to assess the impact of an undergraduate Global Health Program on students, alumni, and international partners.

**Methods:**

REM, a new, innovative, community-centered research methodology was employed in this research study whereby three REM focus group sessions, each with 10–11 participants, were facilitated. A multi-layered textual, thematic analysis was used to analyze the data collected from REM focus group sessions.

**Findings:**

After analysis, six thematic areas emerged, each with their own underlying qualities of growth, or sub-themes, which provide insight into the manner in which the major themes contributed to student learning. Furthermore, programmatic components were identified, which aided student growth and learning.

**Conclusions:**

Findings suggest that the undergraduate Global Health Program has promoted and facilitated student growth and learning in various capacities. This study fills a gap in existing research and current knowledge by outlining the impact of an undergraduate Global Health Program on students. Additional studies should be conducted to further explore the impact of Global Health Programming on students and stakeholders.

## Background

Over the last decade, both in the US and internationally, there has been a dramatic rise in the interest in global health as an academic discipline [1]. More students at both the undergraduate and graduate levels are now pursuing academic global health opportunities, but little evaluative research exists on the impact of such experiences. Global Health is a collaborative discipline that involves partnership and capacity building for both high- and low-income partners [2]. During the 2019–2020 academic year, 84 colleges and universities offered minors in global health [3].

In 2006, Cornell University received a National Institutes of Health grant to develop an undergraduate Global Health Program (GHP). With this grant, a university wide minor in Global Health was created in 2007. Due to student demand, a major in Global and Public Health Sciences was offered beginning in 2014. Since 2007, over 560 students from diverse academic disciplines have completed the minor and 124 students have graduated with the major. Experiential learning in global and public health is a requirement for both majors and minors. Students can develop their own Experiential Learning Opportunity (ELO) or participate in a GHP partnership in India, Tanzania, or Zambia. In the longest standing partnership, faculty from Cornell University and Kilimanjaro Christian Medical University College (KCMUCo) have worked together for over a decade to design, implement, and evaluate the KCMUCo-Cornell Collaborative Program, an engaged learning program in global health and development policy [4]. These partnerships and programs create rich opportunities for engaged learning, research, and community service in global and public health.

Beyond ELOs, students continue to engage in global health programming while on the University’s campus. The GHP has created student leadership opportunities such as teaching assistantships and student advisory board positions that allow student leaders to participate in program development and curriculum innovation. Additionally, the program has integrated innovative pedagogy into the classroom incorporating group work, poetry, and art into educational experiences. Finally, connections are cultivated with students throughout the undergraduate experience, providing mentorship opportunities at every stage of the student’s career.

In recent years, there has been renewed scrutiny of global health and calls for an examination of the pervasive inequities in research and practice [5,6,7,8,9]. Despite the burgeoning growth of global health programs and curricula in: high-income countries over the past decade, the volume of research evaluating global health training programs and partnerships is limited. While there is a growing body of research on global health clinical training and education in medical schools, the impacts and outcomes of global health programming at the undergraduate level are yet to be fully examined and documented [10,11].

Since its inception, the GHP has used continuous developmental evaluation strategies, such as formal course evaluations, peer feedback tools, team debriefs, and reflection sessions to improve curriculum, engaged learning programs, and student learning outcomes. In order to better understand the long-term impact of the program, Ripple Effects Mapping (REM) methodology was utilized. REM is an innovative qualitative methodology utilized to examine the impacts of collaborative educational and community programs. In the past, REM has been utilized to evaluate academic-community partnerships as well as community-based health programs [12,13]. In this article, REM is used to evaluate an educational program. Reflective focus group sessions and interviews are used to collect in-depth stories as data from participants. REM sessions consist of eight to 12 participants [14]. A unique feature of REM is that it allows researchers to gather direct feedback through the personal lens of participants, which can inform program improvement and development [14]. Mind maps are an additional tool used to highlight the thematic ripples and connections between participant stories.

REM is grounded in four core principles; (1) appreciative inquiry, (2) a participatory approach, (3) interactive group interviewing and reflection, and (4) mind mapping [14]. Appreciative inquiry incorporates questions developed by the researchers that guide participants through reflection on key aspects of their program experience in pairs. Facilitated appreciative inquiry gives participants time to reflect on personal experiences and incorporates a positive approach to brainstorming stories [14]. It is an asset-based, positive storytelling approach. A participatory approach is utilized throughout REM to move beyond thinking of program stakeholders as “recipients” but rather as a part of the evaluation [14]. During the REM sessions, interactive group interviewing and reflection allows for the facilitator and participants to work together to generate mind maps. Mind maps are data visualization tools that aid in illustrating the “chain of events” of a program or project [14].

The purpose of this qualitative study was to evaluate the impact of the GHP on students, alumni, and partners and identify key areas of student development and integral curricular components utilizing the REM methodology.

## Methods

### Study Design

This qualitative study utilizing REM methodology consisted of three focus group sessions facilitated by trained REM staff. Each session lasted approximately two hours.

At the beginning of each session, participants were invited to develop and write a personal story based on the following question: “Tell us a story about how being involved in the Global Health Program has impacted you.” After this stage, participants came together in pairs to share their written stories through appreciative inquiry. In the next stage, participants came together as a group to share their individual stories. At this point, facilitators documented the stories using mind maps, which aided in the identification of ripples and themes.

**Figure 1:**
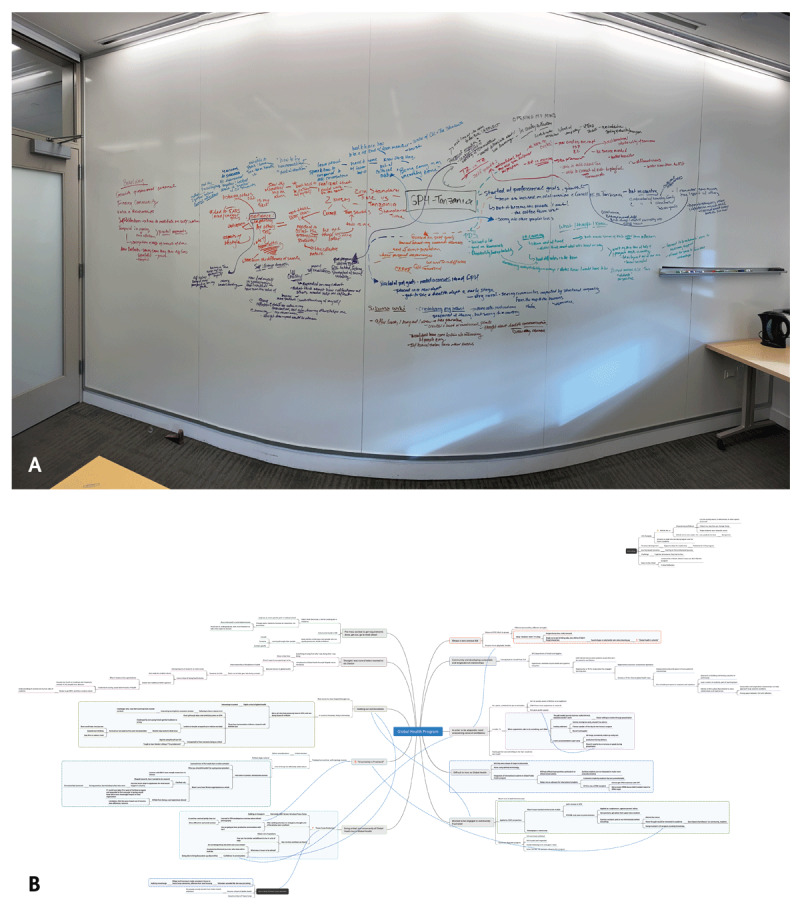
Mind Map examples created during different REM sessions (A is a visual map, B is a digital map). Each are of different REM sessions. **A:** Visual Map. **B:** Digital Map.

Mind Mapping was done using two strategies: visual maps and digital maps. A visual map was drawn in real time during the REM group session so that it was visible to all participants (Figure 1a). This was either on a white board or large piece of poster paper. A digital map was simultaneously logged by a member of the research team using Mind Mapping software (Xmind) (Figure 1b). This digital map was not shared with session participants but used for cross-comparison with the visual map.

All sessions began by creating space for openness and dialogue. Food was provided and there was downtime before beginning the session. Three research roles were needed during the REM session. The primary facilitator led the group through the storytelling process and drew the visual map. A second facilitator documented the digital map using XMind software. The final researcher took detailed notes during the session. All sessions were audio recorded and transcribed after informed consent from all participants was acquired. IRB approval from Cornell University (#1910009157) was sought and the study was declared exempt because it was a program evaluation.

### Sample

REM criteria for selecting a sample is intentional—samples are meant to incorporate individuals who have a deep engagement within the program being evaluated. Intentionally selecting participants who have been deeply involved in the named program allows for more comprehensive data to be gathered [9]. Inclusion criteria for this study consisted of participants who had a high level of sustained engagement with the GHP. In this study, sustained engagement was defined as students and alumni who had actively been involved in more than one component of the GHP. This includes serving as a leader in various capacities, such as a teaching assistant, student advisory board member, and international program and partnership assistant.

Ten to 11 participants were selected for each group. For this evaluation, three REM focus group sessions were conducted. The sessions were as follows: student leaders (current), past Cornell participants from the KCMUCo-Cornell Collaborative Program, and alumni of the GHP. Focus group sessions consisted of a total of 31 unique individuals.

Ten unique participants engaged in the Student Leaders group consisting of current program leaders, all of whom were current third- or fourth-year undergraduate students majoring in Global and Public Health Sciences or minoring in Global Health at the time of the focus group session.

Ten unique participants engaged in the KCMUCo-Cornell Collaborative Program group, which consisted of current third- and fourth-year undergraduate students, as well as program alumni (graduates of Cornell University). There were four current undergraduate students and six alumni in this group. The criteria for group eligibility included that the participant must have participated in the KCMUCo-Cornell Collaborative Program in Moshi, Tanzania during their undergraduate experience.

The last group, the “Alum Group,” consisted of 11 unique participants who were current University and GHP alumni. All alumni either majored in Global and Public Health Sciences or minored in Global Health during their undergraduate career.

### Data Analysis

Qualitative thematic content analysis was completed by a team of three researchers. The researchers completed analysis in five stages. In stage one, the researchers reviewed REM focus group session and interview notes alongside the visual and digital (XMind) maps. This stage was used to compile field notes and remark on general themes. As a part of stage two, in order to ensure correct thematic analysis, two follow-up individual in-depth interviews were done with student leaders in the program. The individual interviews clarified themes that were beginning to arise. In stage three, the research team conducted a multi-layered textual analysis, using full transcripts of group sessions and interviews to elucidate six major themes and additional sub-themes, also known as qualities of growth, corresponding to each respective theme. Qualities of growth are the areas of academic, professional and personal development and transformation that allow an individual to achieve the overarching theme (Figure 2.1–6 listed below each theme). In stage four, the transcripts were used to develop codes for each individual story which allowed stories to be categorized by overarching theme and the quality of growth. The research team tracked the number of times each overarching theme was highlighted in individual student stories shared during REM sessions. Quotes were also identified that corresponded to each theme. Additionally, programmatic components highlighted in participant stories were distinguished and identified in this stage. Programmatic components are the core courses, requirements, student-centered development opportunities, and integrative elements of the GHP. Each programmatic component was defined based on the student stories. The programmatic components mentioned in REM focus group sessions were tallied. In stage five, the research team compared the research findings to existing frameworks for Global and Public Health undergraduate students.

**Figure 2:**
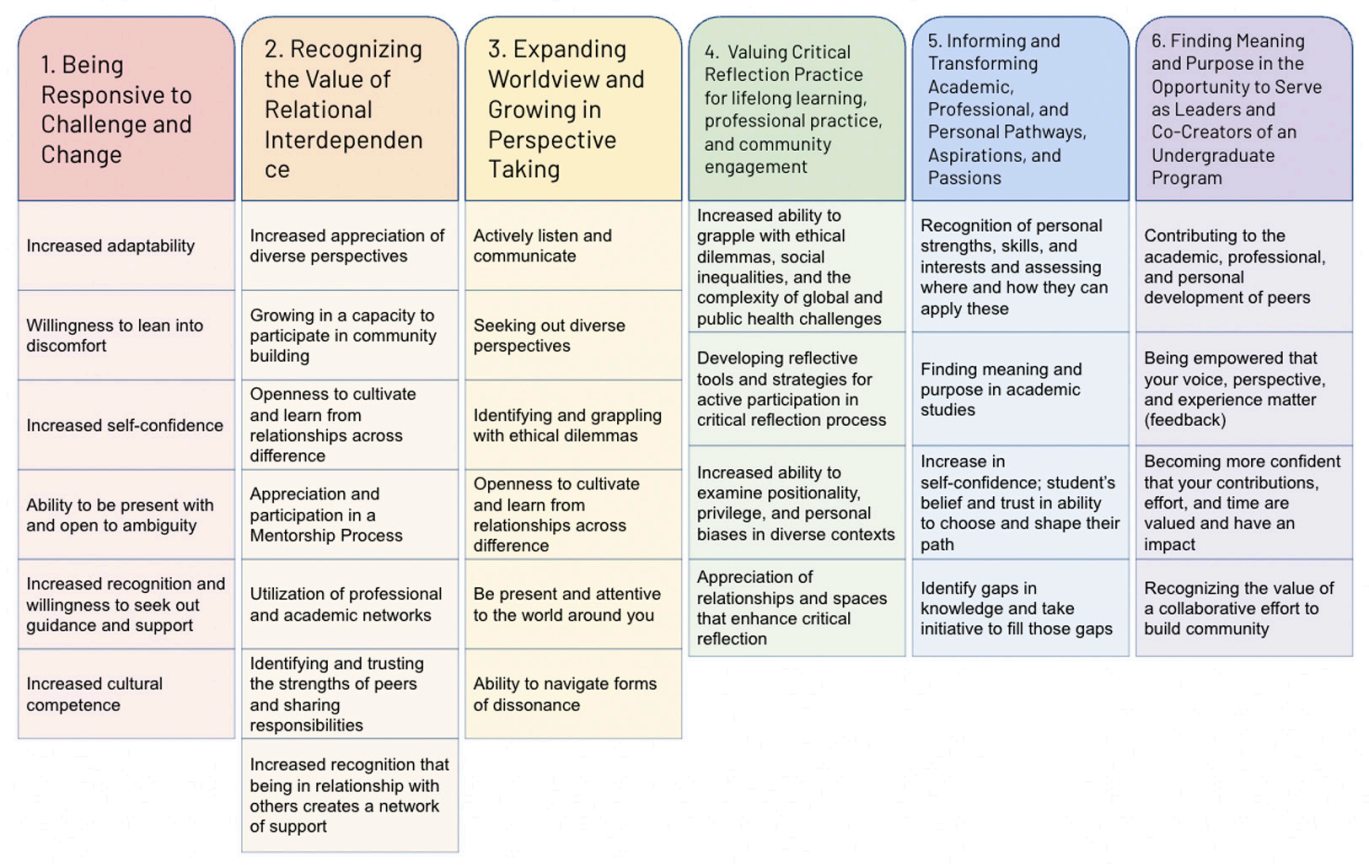
Six major themes with underlying qualities of growth.

## Results

After textual analysis, six major themes (Figure 2) emerged from the data, each with their own set of sub-themes, which we call, in this article, “qualities of growth” (Figure 2.1–6). Each individual theme had four to seven distinct qualities of growth. Frequently, these themes and qualities of growth were mentioned in relation to specific programmatic components. A total of 22 programmatic components arose from sessions. Programmatic components mentioned by more than 25% of REM session participants are defined and highlighted in this article (Table 1).

**Table 1:** Programmatic components, definitions, and the frequency that they arose from student stories. Only components that were in over 25% of student stories are featured.


PROGRAMMATIC COMPONENT	DEFINITION	PERCENTAGE OF PARTICIPANTS WHO IDENTIFIED THIS COMPONENT

Experiential Learning Opportunity (ELO)	A core curricular requirement that provides the opportunity for students to integrate academic knowledge, conceptual tools, and skills from coursework in an applied global and public health experience.	81% (n = 25)

“Global” Partners and Relationships	Partnerships with local and global stakeholders, including host families, colleagues in engaged learning placements, and international program staff, create opportunities for students to be both challenged and mentored in new settings.	61% (n = 19)

Near Peer Relationships	Engaged learning programs allow students from diverse backgrounds to develop friendships, collaborations, and networking connections through facilitated opportunities to live, learn, and work together.	58% (n = 18)

Critical Reflection	Critical reflection practice is integrated throughout the curriculum with written assignments, cross-cultural dialogues, collaborative projects, and on/off campus activities to guide students in developing critical reflection skills.	52% (n = 16)

Cohort Community Building	Creation of intentional spaces for groups of students to collaboratively learn and work together, experience and respond to challenges, contribute to program development, and form personal and professional relationships.	45% (n = 14)

Teamwork	Multi-faceted opportunities for students to work towards a common goal with their peers. Teamwork is integrated throughout curricular requirements, international partnerships, engaged learning and research, and leadership opportunities.	42% (n = 13)

Dialogue Across Difference [15]	Collaborative communication between individuals with unique, diverse perspectives within and a part of the GHP. This includes peers, stakeholders, faculty/staff, and program partners.	42% (n = 13)

Mentorship	Guidance and support provided by faculty, staff, local and global partners, and other mentor figures that work directly with our students in a professional setting.	39% (n = 12)

Student Leadership Opportunities	Embracing and utilizing student knowledge, lived experience, and the passion and commitment to sustain and strengthen programmatic partnerships, curriculum, and activities. Student leaders are invited to participate in all parts of the GHP, ranging from the advisory board to program and teaching assistant positions.	26% (n = 8)


### Theme 1: Being Responsive to Challenge and Change

The first theme was “Being Responsive to Challenge and Change,” with six emergent qualities of growth (Figure 2.1), including: increased adaptability, willingness to lean into discomfort, increased self-confidence, ability to be present with and open to ambiguity, increased recognition and willingness to seek out guidance and support, and increased cultural competence. The theme emphasizes that through their participation in the GHP, students developed an increased ability to respond and react to challenging situations in life and in work. Cohort community building was the programmatic component that was most frequently identified as facilitating opportunities to be responsive to challenge and change. This theme was featured in 26% of student stories (n = 8).

*“I felt that I was always goal oriented, but I don’t think I had the patience to pursue those things. We ran into a lot of obstacles where I was really put on the spot in certain situations and in those times, I realized that what I needed the most to solve the issue was to be more patient with myself.” (KCMUCo-Cornell Collaborative Program group participant)*

*“… [I gained an ability to be] comfortable with being uncomfortable in a lot of situations.” (Alumni group participant)*

### Theme 2: Recognizing the Value of Relational Interdependence

The second theme was “Recognizing the Value of Relational Interdependence,” with seven emergent qualities of growth (Figure 2.2), including: increasing appreciation of diverse perspectives; growing in a capacity to participate in community building; being open to cultivating and learning from relationships across differences; appreciating and participating in a mentorship process; utilizing professional and academic networks; identifying and trusting the strengths of peers and sharing responsibilities; and increasing recognition that being in relationship with others creates a network of support. Together, these qualities of growth illustrate an appreciation of and desire to collaborate with and learn from diverse individuals in various contexts. Near peer relationships, cohort community building, and teamwork were the programmatic components most frequently identified as promoting recognition of the value of relational interdependence. This theme was featured in 68% of student stories (n = 21).

*“I may not be the person with the loudest voice or the one that is the most charismatic leader, but I really found my niche in listening to others, hearing others out and bringing everyone together. That is really something I learned about myself in engaging in this very elaborate teamwork setting, and I don’t think I would have been able to gain that from any other teamwork environment I was exposed to at [The University].” (KCMUCo-Cornell Collaborative Program group participant)*

*“In this setting, since I didn’t speak the language, I wasn’t the best person for the job, I really had to learn how to be okay with that. Working in a team doesn’t mean you have to be the lead person all the time. Understanding the strengths of other people was much more useful in this setting.” (KCMUCo-Cornell Collaborative Program group participant)*

### Theme 3: Expanding Worldview and Growing in Perspective Taking

The third theme was “Expanding Worldview and Growing in Perspective Taking,” with six qualities of growth (Figure 2.3), including: actively listening and communicating, seeking out diverse perspectives, identifying and grappling with ethical dilemmas, openness to cultivate and learn from relationships across difference, being present and attentive to the world around you, and the ability to navigate forms of dissonance. The qualities of growth emphasize a deepening of participants’ capacities to engage diverse perspectives, articulate their worldview, and navigate forms of dissonance. The ELO, global partners and relationships, and dialogue across differences were the programmatic components most frequently identified as facilitating expansion of worldview and growing in perspective taking. This theme was featured in 29% of student stories (n = 9).

*“I started to ask myself a lot of questions about my path. I guess by understanding different people and their backgrounds and how they live their life, I started to reflect a lot on my path.” (KCMUCo-Cornell Collaborative Program group participant)*

### Theme 4: Valuing Critical Reflection Practice for Lifelong Learning, Professional Practice, and Community Engagement

The fourth theme was “Valuing Critical Reflection Practice for lifelong learning, professional practice, and community engagement,” with four qualities of growth (Figure 2.4), including: increasing ability to grapple with ethical dilemmas, social inequalities, and the complexity of global and public health challenges; developing reflective tools and strategies for active participation in critical reflection process; increasing ability to examine positionality, privilege, and personal biases in diverse contexts; and appreciating relationships and spaces that enhance critical reflection. This theme emphasizes students’ growing ability to critically reflect through the use of new tools, strategies, and relationships. Critical reflection was the programmatic component most frequently identified as facilitating opportunities for students to value critical reflection practice for lifelong learning, professional practice, and community engagement. This theme was featured in 26% of student stories (n = 8).

*“I think there is so much room for critical reflection and how it [has] shaped you and those around you and so I think that’s a very valuable principle of the program.” (Student Leaders Group Participant)*

*“Learning that there is more than what we see, being able to take time and talk to people, building that trust with your patients and with whoever you interact with and thinking of them as a whole person instead of just the symptoms that they show. A lot of times people forget that. That is something I carry forward [from the Global Health Program] as I practice in my own career.” (Alumni group participant)*

### Theme 5: Informing and Transforming Academic, Professional, and Personal Pathways, Aspirations, and Passions

The fifth theme was “Informing and Transforming Academic, Professional, and Personal Pathways, Aspirations, and Passions”, with four qualities of growth (Figure 2.5), including: recognizing personal strengths, skills, and interests and assessing where and how they can apply; finding meaning and purpose in academic studies, and increasing self-confidence; believing and trusting in the ability to choose and shape their path; and identifying gaps in knowledge and taking initiative to fill those gaps. This theme highlights the growing capacity of participants to pursue and identify their passions and interests in global and public health. The ELO and mentorship were the programmatic components most frequently identified as important for transforming academic, professional, and personal pathways. This theme was featured in 48% of student stories (n = 15).

*“It feels really great for me to have a deeper understanding of why I want to be in the health field and why I want to go into medicine.” (KCMUCo-Cornell Collaborative Program group participant)*

*“[The ELO] becomes a turning point in a lot of people’s lives and stories. It’s hard to replicate that kind of life changing experience and it’s actually amazing that it’s replicable as many times as it is.” (Alumni group participant)*

### Theme 6: Finding Meaning and Purpose in the Opportunity to Serve as Leaders and Co-Creators of an Undergraduate Program

The sixth theme was “Finding Meaning and Purpose in the Opportunity to Serve as Leaders and Co-Creators of an Undergraduate Program”, with four qualities of growth (Figure 2.6), including: contributing to the academic, professional, and personal development of peers; being empowered that your voice, perspective, and experience matter (feedback); becoming more confident that your contributions, effort, and time are valued and have an impact; and recognizing the value of a collaborative effort to build community. These qualities of growth demonstrate the value and empowerment that students experience when invited to be part of a larger team and mission. Student leadership opportunities was the programmatic component most frequently identified as facilitating opportunities to find meaning and purpose in the opportunity to serve as leaders and co-creators of an undergraduate program. This theme was featured in 13% of participants’ stories (n = 4).

*“Being able to lead a cohort of students and being a part of the teaching team allowed me to have that sense of community within the overall global health community here at Cornell. And it really allowed me to value those relationships and value that experience.” (Student Leader group participant)*

## Discussion

This qualitative study demonstrated the different areas of student personal and professional growth facilitated by the GHP. The study demonstrated that students value experiential learning opportunities, relationship building, being challenged, learning critical reflection practices, and the opportunity to contribute to their own educational and professional experiences. Additionally, this study demonstrated the successful use of REM methodology in evaluating such a program.

An exceptional finding is the link between programmatic components and the personal and professional success of students. Participant stories highlighted the ways that specific programmatic components such as the “Experiential Learning Opportunity (ELO)” directly contributed to themes and qualities of growth. Some participants connected the ELO to professional pathways and careers later followed. As results indicate, 81% of participants mentioned the Experiential Learning Opportunity during REM sessions, indicating its particular significance in defining their experience in the GHP. Such an experience should be considered when implementing global health programs at the undergraduate level. Additionally, 61% of participants mentioned “Global Partners and the Value of Relationships,” also indicating the significance of such components to academic and enriching programs. This suggests that emphasizing relationship building in global health programs should be core to program implementation. The programmatic components breakdown could be used to guide future academic undergraduate global health programs striving to build successful student leaders (Table 1).

Further, results from this study suggest that the GHP allowed participants to grow in the six thematic areas outlined (Figure 2.1–6), indicating the program could have long-term impacts on personal and professional growth. The qualities of growth, or sub-themes, provide additional insight into the manner in which the major themes contributed to student learning. Additional research should be done on existing global health programming at other institutions in order to further validate these thematic areas.

The findings from this study were compared to existing frameworks in Global and Public Health education. One framework, the Association of Schools and Programs in Public Health, highlights four competencies—all of which overlapped with the findings presented in this research (Knowledge of Human Cultures and the Physical and Natural World as it Relates to Individual and Population Health, Intellectual and Practical Skills, Personal and Social Responsibility, and Integrative and Applied Learning) [16]. Another framework from the Consortium of Universities for Global Health (CUGH) highlights 11 competency domains, with which this research has many overlapping areas [17]. For example, the CUGH competency “Collaboration, Partnering and Communication” aligns well with Theme 2: Recognize the Value of Relational Interdependence. When compared to these frameworks, the findings from this study reveal that students are achieving desired global and public health competencies and learning goals.

REM was a successful strategy to evaluate a global health program. The use of this new, innovative methodology allowed for participants to have a voice in data analysis and celebrate the program and their own growth as individuals. Participants enjoyed taking part in the sessions and found meaning in participating directly in the evaluation process. The analysis of REM visual and digital maps (Figure 1a–b) proved to be difficult. The visual maps were primarily utilized for visualization by the participants during the actual REM session. The digital (Xmind) map was referred to as needed by the research team. Additional analysis using data transcription of REM sessions proved more effective for coding purposes.

This evaluation assessed the impact of a long term undergraduate global health program and documented the transformative impact and key programmatic components. It begins to fill existing gaps in knowledge around the influence of global and public health programs at the undergraduate level. The results provide insights into the critical components of undergraduate global health programming needed to meaningfully engage and challenge students to make connections between theory and practice, acknowledge diverse forms of dissonance, confront bias and inequities and be active participants in their own learning. This study also reveals the utility of the REM methodology for elucidating the unique ways that students are learning and growing through their engagement in global and public health education.

### Limitations

A potential limitation is confirmation bias, which could have existed in the analysis of the REM sessions by study team evaluators. The evaluators of the program were deeply involved in the development and implementation of many facets of the programming. This is due to the limited human resource capacity, including a lack of professional employees, an ongoing challenge for the GHP. A positive outcome of this challenge has been robust student leadership opportunities, whereby student leaders are involved in every aspect of the program ranging from curriculum development to program evaluation activities. Due to their high level of involvement, two members of the research team were also participants in the evaluation. To minimize the potential for bias, the research team was highly conscientious of this risk in conducting the analysis. For this reason, the research team documented the sessions in multiple formats, including: transcripts, digital and visual mind maps, and notes taken during REM sessions that were incorporated into the multi-layered analysis.

Another potential limitation is that the sample was constrained by geographical distance and availability to attend the sessions in person. Although there were many eligible participants based on their level of commitment and engagement to the GHP, only those participants that could access the location of the session were included. While this type of convenience sampling could contribute to potential biases, this type of purposeful sampling is essential to the REM methodology. For this reason, the researchers hosted three separate REM sessions hoping to gather more data in order to limit potential biases [14]. Due to the COVID-19 pandemic, research plans to conduct REM sessions with international partners and program alumni in Tanzania were delayed. Future work by the research team will address this gap.

REM is just one manner in which to evaluate the impact of global health programming and education on undergraduate students. Additional studies using various methodologies should be conducted in order to fill the gap in knowledge on the impact of programming in global health education.
